# Instruction of Circulating Endothelial Progenitors *In Vitro* towards Specialized Blood-Brain Barrier and Arterial Phenotypes

**DOI:** 10.1371/journal.pone.0084179

**Published:** 2014-01-02

**Authors:** Julie Boyer-Di Ponio, Fida El-Ayoubi, Fabienne Glacial, Kayathiri Ganeshamoorthy, Catherine Driancourt, Maeva Godet, Nicolas Perrière, Oriane Guillevic, Pierre Olivier Couraud, Georges Uzan

**Affiliations:** 1 Inserm U972, Hôpital Paul Brousse, Villejuif, France; 2 ABCell-Bio, Paris, France; 3 Inserm U1016, Institut Cochin, Paris, France; 4 Cnrs, UMR8104, Paris, France; 5 Université Paris Descartes, Sorbonne Paris Cité, Paris, France; 6 VigiCell - Villejuif Biopark, Villejuif, France; Biological Research Centre of the Hungarian Academy of Sciences, Hungary

## Abstract

**Objective:**

The vascular system is adapted to specific functions in different tissues and organs. Vascular endothelial cells are important elements of this adaptation, leading to the concept of ‘specialized endothelial cells’. The phenotype of these cells is highly dependent on their specific microenvironment and when isolated and cultured, they lose their specific features after few passages, making models using such cells poorly predictive and irreproducible. We propose a new source of specialized endothelial cells based on cord blood circulating endothelial progenitors (EPCs). As prototype examples, we evaluated the capacity of EPCs to acquire properties characteristic of cerebral microvascular endothelial cells (blood-brain barrier (BBB)) or of arterial endothelial cells, in specific inducing culture conditions.

**Approach and Results:**

First, we demonstrated that EPC-derived endothelial cells (EPDCs) co-cultured with astrocytes acquired several BBB phenotypic characteristics, such as restricted paracellular diffusion of hydrophilic solutes and the expression of tight junction proteins. Second, we observed that culture of the same EPDCs in a high concentration of VEGF resulted, through activation of Notch signaling, in an increase of expression of most arterial endothelial markers.

**Conclusions:**

We have thus demonstrated that *in vitro* culture of early passage human cord blood EPDCs under specific conditions can induce phenotypic changes towards BBB or arterial phenotypes, indicating that these EPDCs maintain enough plasticity to acquire characteristics of a variety of specialized phenotypes. We propose that this property of EPDCs might be exploited for producing specialized endothelial cells in culture to be used for drug testing and predictive *in vitro* assays.

## Introduction

According to their specific functions, different organs establish during development instructive interactions with the circulatory system, leading to the generation of blood vessels displaying different specific features. Depending upon genetic predisposition and microenvironmental influences, immature endothelial cells have the ability to differentiate into specialized subpopulations with gene expression profiles characteristic of different vascular types [1]–[3]. The post-natal vascular system is thus heterogeneous, and depending on their anatomic localisation, endothelial cells have acquired specialized functions. Some endothelial cells like human umbilical vein or artery endothelial cells (HUVECs or HUAECs), are easy to isolate, but others, such as brain endothelial cells are not. Moreover, when isolated and cultured, these specialized mature endothelial cells rapidly lose their specific differentiated features after only a few passages, limiting the usefulness of these cultures for producing predictive and reproducible pharmacological models.

Circulating endothelial progenitor cells (EPCs) are mobilized from the bone marrow and are able to integrate into vascular structures at sites of neovascularisation where they differentiate into endothelial cells and proliferate [4], [5]. Many studies suggest that the physiological function of EPCs is the maintenance of vascular integrity [6]–[10]. Cord blood EPC-derived endothelial cells (EPDCs) isolated from human umbilical cord blood can be extensively expanded *in vitro*. When compared to mature vessel endothelial cells, such as HUVECs, EPDCs express endothelial markers to a similar extent, except for KDR, which is expressed at a higher level in EPDCs [11]. Moreover, they displayed a higher proliferation potential. Functional studies also have demonstrated that these cells are more sensitive to angiogenic factors, conferring to EPDCs a better viability than HUVECs [11]. Furthermore, compared to equivalent cells isolated from adult peripheral blood, those from cord blood give rise to a higher number of colonies. Moreover, only cord blood EPCs, when seeded under limiting dilution conditions, give rise to secondary and tertiary colonies: these observations suggest that cord blood EPCs still display properties of immature cells [12], [13]. In this study, we explore the hypothesis that these cells might be induced to acquire features of distinct specialized endothelial cells *in vitro* when exposed to appropriate external instructive stimuli. Because such specialization might be of considerable interest for the development of new *in vitro* models for drug testing, we focused the present study on two distinct endothelial specialized phenotypes with potential pharmacological value: the blood brain barrier (BBB) and arterial phenotypes.

The BBB is responsible for strictly controlling the exchanges between the blood and brain compartments, by preventing the paracellular diffusion of hydrophilic solutes, mediating the active transport of nutrients to the brain, effluxing hydrophobic molecules and drugs from the brain to the blood and regulating the trans-endothelial migration of circulating blood cells and pathogens. Endothelial cells of cerebral blood vessels display a unique phenotype characterized by the presence of intercellular tight junctions and the polarized expression of numerous transport systems [14], [15]. In close proximity to brain ECs, pericytes, glial cells (especially astrocytes), and neurons, together with the basement membrane sheathing cerebral blood vessels, are directly involved in the establishment and maintenance of the BBB. Most *in vitro* BBB models currently available are based on primary cultures of cerebral endothelial cells from different animal (bovine, porcine, murine cells) and human sources, usually co-cultured with glial cells in two-chamber cell culture systems [16]–[19]. In addition, we previously developed the human hCMEC/D3 brain endothelial cell line, which retains many morphological and functional characteristics of brain endothelium, as a tentative *in vitro* model of the human BBB [20]–[22]; indeed, this model has now been validated and is widely used as a model of brain endothelium. However, because of the limitations of this model, in particular its relatively high paracellular permeability to small hydrophilic compounds, there is still a real need to develop new models of human BBB, based on primary culture of specialized endothelial cells, both for studying mechanisms involved in BBB specialization and for performing reliable and predictive pharmacological and toxicological tests.

Specification of endothelial cells to an arterial or venous fate is a crucial process during vascular development. With the discovery of ephrin B2 (Efnb2) and its receptor ephrin B4 (Ephb4) as biomarkers of arteries and veins, respectively, it appeared that the specification of arteries and veins is determined by genetic programs in the developing embryo before the appearance of the circulation [23]. It has been shown that a high dose of VEGF induces arterial specification by controlling Notch pathway induction [24]. Numerous studies indicate that activation of Notch signalling is essential to arterial specialization [25], [26]. Most studies which focused on the crucial role of signalling cascades such as VEGF and Notch in arterial specification have been performed on mouse embryonic stem cells [24], [27], [28]. A recent study characterized the flow-induced transcriptional response of EPCs. It showed that these cells do not resemble mature arterial cells in their expression of specific differentiation markers [29]. These results confirm that blood flow alone is not sufficient for arterial specialization.

In the present study, we demonstrate that human cord blood EPCs retain an immature phenotype, allowing them to further differentiate *in vitro,* upon instruction by appropriate stimuli, towards various specialized phenotypes such as BBB or arterial endothelial phenotypes.

## Materials and Methods

### Cell Isolation and Culture

Mononuclear cells (MNCs) were isolated from human cord blood (all anonymous donors gave informed consent for all procedures and samples) by Ficoll (Pancoll, Dutscher, France) density gradient centrifugation (400 g, 30 min, 20°C, no frame) and were resuspended in endothelial growth medium (EGM2-MV) (Lonza, Verviers, Belgium) [9]. The cells were then plated at a cell density of 5.2×10^6^/cm^2^ onto separate wells of a 12-well tissue culture plate precoated with type I rat tail collagen (BD Biosciences, Le Pont de Claix, France) and maintained at 37°C, under 5% CO2, in a humidified incubator. In order to eliminate non-adherent cells and debris, the medium was aspirated after 24 hours of culture, wells were washed with PBS 1X and fresh complete EGM-2 medium was added to each well. The medium was changed daily for 7 days and then every other day. Wells were inspected daily for the outgrowth of endothelial colony-forming cells (ECFC). ECFC colonies appeared between 8 and 12 days of culture and were characterized by formation of a cluster of cobblestone-appearing cells.

Human samples were collected and handled in the full respect of the declaration of Helsinki. Cord blood used for endothelial cells preparation is managed through partnership with The Cord Blood Bank of St Louis Hospital. This cord blood bank is authorized by French Regulation Agency (authorization N° PPC51) and participates to scientific research. This activity has been declared to and authorized by French Ministry of Research under number AC-2008-376, and to the French Normalization Agency under number 201/51848.1. The mothers’ written informed consents are kept by the mother, the maternity administration and the Cord Blood Bank of St Louis Hospital.

HUVECs and HUAECs were isolated according to the method of Jaffe et al. [30] and cultured in EGM2-MV. hCMEC/D3 were established as described [21] and cultured in EBM2 medium (Lonza) supplemented with 5% FBS “Gold” (PAA Laboratories GmbH, A15–151), 10 mM HEPES (PAA Laboratories GmbH), 1% Penicillin-Streptomycin, 1% chemically defined lipid concentrate (Invitrogen Ltd, Paisley, UK), 1.4 µM hydrocortisone, 5 µg/ml ascorbic acid, 1 ng/ml bFGF (Sigma-Aldrich, St. Louis, MO) and 10 mM lithium chloride (Merck). Human aortic endothelial cells (HAECs) were purchased from ScienCell™ Research Laboratories (cat. 6100, San Diego, CA) and cultured in EGM2-MV. Primary cultures of astrocytes were prepared from the cerebral cortex of newborn rats as previously described [31]. Three weeks after seeding, astrocytes were trypsinized and frozen in liquid nitrogen.

### Flow Cytometry

EPDCs were detached with trypsin and immunophenotyping was assessed by using the following monoclonal antibodies: CD31-FITC (1∶25, BD Pharmingen, 555445), CD144-PE (1∶10, Beckman Coulter, A07481), anti-KDR-APC (1∶5, R&D Systems, FAB357A). Antibodies and matched isotype control (Beckman Coulter) were incubated for 30 min at 4°C. Viability was assessed with 7-AAD (Becton Dickinson). Data were acquired and analyzed on a five-parameter flow cytometer (FACScalibur, Becton Dickinson, San Jose, CA) with Weasel software (WEHI, Melbourne, Australia).

### Immunofluorescence Staining

Cells were fixed in 4% paraformaldehyde/PBS for 10 minutes at room temperature and rinsed with PBS 1X. For intracellular staining, cells were permeabilized with 0.1% Triton X100/PBS for 10 minutes at room temperature. Cells were incubated over night at +4°C with antibodies anti-CD144 (1∶200, Beckman Coulter, IM1597), anti-CD31 (1∶20, BD Pharmingen, 550389), anti-ZO1 (1∶200, BD Biosciences, 610966), anti-CL3 (1∶100, Abcam, C0144), anti-CL5 (1∶100, Invitrogen, 34–1600), anti-OCCL (1∶100, Invitrogen, 33–1500), anti-EFNB2 (1∶10, R&D Systems, AF467), anti-NRP1(1∶20, R&D Systems, AF3870), anti-HEY2 (1∶50, Santa Cruz Biotechnology, sc-28747), anti-ANGPT2 (1∶20, R&D Systems, AF623), anti-CXCR4 (1∶100, Abcam, ab2074), anti-COUP-TFII (1∶100, R&D systems, PP-H7147-10), anti-EPHB4 (1∶10, R&D systems, AF3038) or anti-NRP2 (1∶40, R&D Systems, AF2215) antibodies diluted in 3% BSA/PBS and, after 5 PBS 1X washes, labeled with secondary antibodies Alexa Fluor 488 IgG (Invitrogen, Cergy-Pontoise, France). Cells were then stained with 2 µg/mL of 40,6-diamidino-2-phenylindole (DAPI) and examined with a DMR fluorescence microscope (Leica, Rueil Malmaison, France) equipped with a CoolSnap HQ2 camera (Photometrics, Tucson, AZ) controlled by MetaVue® Analyzing Software (Molecular Devices LLC, Sunnyvale, CA).

### Vascular Tube Formation

Twelve-well plates were coated with Matrigel (BD Biosciences, San Diego, CA). EPC-derived cells were detached by trypsin-EDTA treatment and seeded onto Matrigel in EGM-2 at a cell density of 2.10^5^ cells per well. EPDCs were incubated at 37°C and viewed after 4 hours by visual microscopy for observation of capillary-like formation.

### Ac-LDL Uptake

Incorporation analysis of acetylated low-density lipoprotein 488 (Ac-LDL 488; Invitrogen) was performed to assess the incorporation ability of EPDC. Cells were incubated with 15 mg/ml of Ac-LDL 488 in endothelial medium for 4 h at 37°C, washed three times in PBS, fixed for 10 minutes with 4% paraformaldehyde and mounted with Glycergel mounting medium (Dako, Trappes, France) containing 2 µg/mL DAPI. Ac-LDL uptake was monitored with a DMR fluorescence microscope (Leica, Rueil Malmaison, France) equipped with a CoolSnap HQ2 camera (Photometrics, Tucson, AZ) controlled by MetaVue® Analyzing Software (Molecular Devices LLC, Sunnyvale, CA).

### Co-culture of Human Endothelial Cells and Rat Astrocytes

Astrocytes were thawed and seeded (8.10^4^ cells/cm^2^) into the bottom of 6-multiwell plates four days before endothelial cell seeding. EPDCs, HUVECs or HAECs at passage 2 were then seeded on collagen IV–fibronectin (0.1 mg/mL and 0.02 mg/mL, respectively)-coated well Millicell inserts (Well Millicell® Hanging Cell Culture Inserts, polyester (PET), pore size 1 µm, ref. PIRP30R48) in complete EGM2-MV (LONZA) medium supplemented with 1.4 µM hydrocortisone (a concentration higher than physiological levels, but previously identified as the optimal concentration for BBB differentiation of brain endothelial cells [32]) at a cell density of 4500 cells/cm^2^. Three or four days later, when endothelial cells reached confluence, half of the medium was replaced by the less proliferative EBM2^+^ medium (EBM2+ FBS+bFGF+hydrocortisone, Lonza). EBM2^+^ medium was not supplemented with VEGF. Half of the medium was changed every 3 days, up to 14 days of culture.

In control wells, endothelial cells were seeded under the same conditions, but without astrocytes.

### Permeability Studies

To measure the paracellular passage of Lucifer Yellow (LY, 457 kDa), a water soluble small fluorescent marker, or of standard fluorescent polar molecules of increasing molecular masses (FITC-Dextran 4 kDa and 70 kDa; Sigma-Aldrich, St. Louis, MO) across the endothelial monolayer, cell culture inserts containing specialized EPDCs, HUVECs or HAECs at passage 2 were transferred into new 6-well plates containing 4.2 mL pre-warmed HBSS/HEPES (1 mM) buffer. At time zero, the culture medium of the upper compartment is removed and replaced by 2 mL of same buffer containing 25 µM of LY, 10 µM FITC-Dextran 4 kDa or 0.25 µM FITC-Dextran 70 kDa molecules. The inserts were transferred at 10, 20, 30, 40 and 60 min to new wells containing the assay buffer. Permeability of LY was also measured on cell-free inserts coated collagen IV–fibronectin. The concentration of the fluorescent marker molecule in samples from the upper and lower compartments was measured with fluorescence multiwell plate reader (excitation: 485 nm, emission: 535 nm). Permeability calculations were made following the clearance principle as described by Siflinger et al. [33] to obtain a concentration-independent permeability value.

### Calcein-AM Uptake

To assess P-gp activity, EPDCs and hCMEC/D3 cells were pre-incubated for 30 min at 37°C in presence or absence of Verapmil (40 µM) (Focus Biomolecules, #10–1111), a P-gp inhibitor. After this pre-incubation, 1 µM of the cell-permeant dye Calcein-AM (AnaSpec, #89202) was added in the medium for 60 min. The cells were then washed 3 times with ice-cold PBS and lyzed on ice with Triton-X100 5% for 20 min. After scrapping and resuspension in a needle, cell extracts were centrifuged for 5 min at 4°C and cellular accumulation of the P-gp substrate Calcein (produced from Calcein-AM after hydrolysis by intracellular esterases) was assessed by fluorescence measurement of the supernatants (excitation: 495 nm; emission: 516 nm) with a Mithras LB 940 (Berthold) spectrophotometer. The P-gp activity is reflected by an increased cellular accumulation of the fluorescent Calcein in presence of the inhibitor Verapamil.

### EPDC Arterial Specialization

To induce arterial specification, EPDCs colonies were trypsinized and then cultured in EGM-2 medium containing 50 ng/mL of VEGF (VEGF-165, PromoKine, C-64420) for one or two passages. Analysis of the expression of arterial and venous markers was then performed. To inhibit arterial differentiation, 50 µM DAPT (Insolution™ γ-Secretase Inhibitor IX, Calbiochem, 565784), a Notch signaling inhibitor, was added to EPDCs at passage 1, during culture in the presence of 50 ng/mL VEGF.

### RNA Isolation and Quantitative Polymerase Chain Reaction

Total RNA of all cell types was isolated using the RNeasy ® Plus Mini Kit (Qiagen, ref. 74134/RNase-Free DNase set, ref. 79254). Following isolation, RNA concentration was quantified using NanoDrop® ND-1000 Spectrophotometer technology. For HUAECs (Human Umbilical Arterial Endothelial Cells) and HUVECs (Human Umbilical Venous Endothelial Cells), cDNA was prepared with the High Capacity cDNA Reverse Transcription Kit (Applied Biosystems, ref. 4368814) using 1 µg of total RNA, and the Quantitative polymerase chain reaction was performed with TaqMan® microfluidic cards (TaqMan Low Density Array: TLDA, Applied Biosystems) on an ABI7900 system at the Service de Génétique Moléculaire, Hôpital du Kremlin-Bicêtre, France. Genes of interest were then selected and results were confirmed with Gene Expression Assays (4331182, Applied Biosystems) using the Taqman Universal MasterMix II (4440047, Applied Biosystems) in an ABI7300 system (Applied Biosystems) in the presence of 10 ng of initial RNA. Accession numbers of TaqMan assays are presented in Table 1. Gene expression was measured using the 2-[/delta][/delta]Ct method. For the hCMEC/D3 cell line, cDNA was prepared with Superscript II Reverse Transcriptase (Invitrogen, ref. 18064014) using between 100 and 150 ng of total RNA, and the quantitative polymerase chain reaction was performed with LightCycler480 Sybr Green I Master® kit (Roche, ref. 04707516001) in a LightCycler 480 system on the genomic platform of the Institut Cochin, France.

**Table 1:** Accession numbers of TaqMan® (Applied Biosystems) assays used for the quantitative-PCR.

Genes	Assay IDs
ANGPT2	Hs01048042_m1
VE-CAD (CD144)	Hs00174344_m1
CD34	Hs00156373_m1
COUP-TFII (NR2F2)	Hs00819630_m1
CXCR4	Hs00976734_m1
DLL4	Hs00174344_m1
EFNB2	Hs00187950_m1
EPHB4	Hs00174752_m1
HES1	Hs00172878_m1
HES2	Hs00219505_m1
HEY1	Hs00232618_m1
HEY2	Hs00232622_m1
JAG1	Hs00164982_m1
KDR (VEGFR2)	Hs00176676_m1
LMOD1	Hs00201704_m1
MMP9	Hs00234579_m1
NOTCH3	Hs00166432_m1
NOTCH4	Hs00270200_m1
NRP2	Hs00187290_m1
PECAM (CD31)	Hs00169777_m1
PLGF	Hs01119262_m1
RPLP0	Hs99999902_m1
SELP	Hs00927900_m1

### Western Blot Analysis

Cell lysates were prepared using 200 µL Laemmli 2X buffer with benzonase, directly on inserts, maintained on ice, for 10 min. After scraping, cell supernatants were harvested and denatured at 100°C for 5 min. Proteins were analyzed on 8% SDS–PAGE gels and blotted on nitrocellulose membranes (Amersham Biosciences). After protein transfer, unspecific binding sites were blocked by incubation in TBS-T (50 mM Tris/HCl, 150 mM NaCl) containing 0.05% Tween 20 and 5% skimmed milk for 1 hour at room temperature. Washed membranes were incubated overnight at 4°C with antibody against P-Glycoprotein C219 (1∶20, Thermoscientific, MA1-26528), occludin (1∶100, Santa Cruz Biotechnology, sc-133255) or Glut-1 (1∶200, Epitomics, 2944S). Four washes were performed in 0.05% TBS-T. Following 1 hour incubation with HRP-conjugated secondary anti-mouse or rabbit antibody (1∶5000 in 2.5% skimmed milk), the target protein was revealed by chemiluminescence HRP substrate (ECL™ Western Blotting Detection Reagents, Amersham Biosciences). Quantification was performed on scanned immunoblot using ImageJ software.

### Statistical Analysis

All data are presented as the mean ± SEM. Comparisons between groups were performed using unpaired student’s t-tests. P values less than 0.05 were considered significant.

## Results

### 1) EPDCs Isolation and Characterization

**Figure 1:**
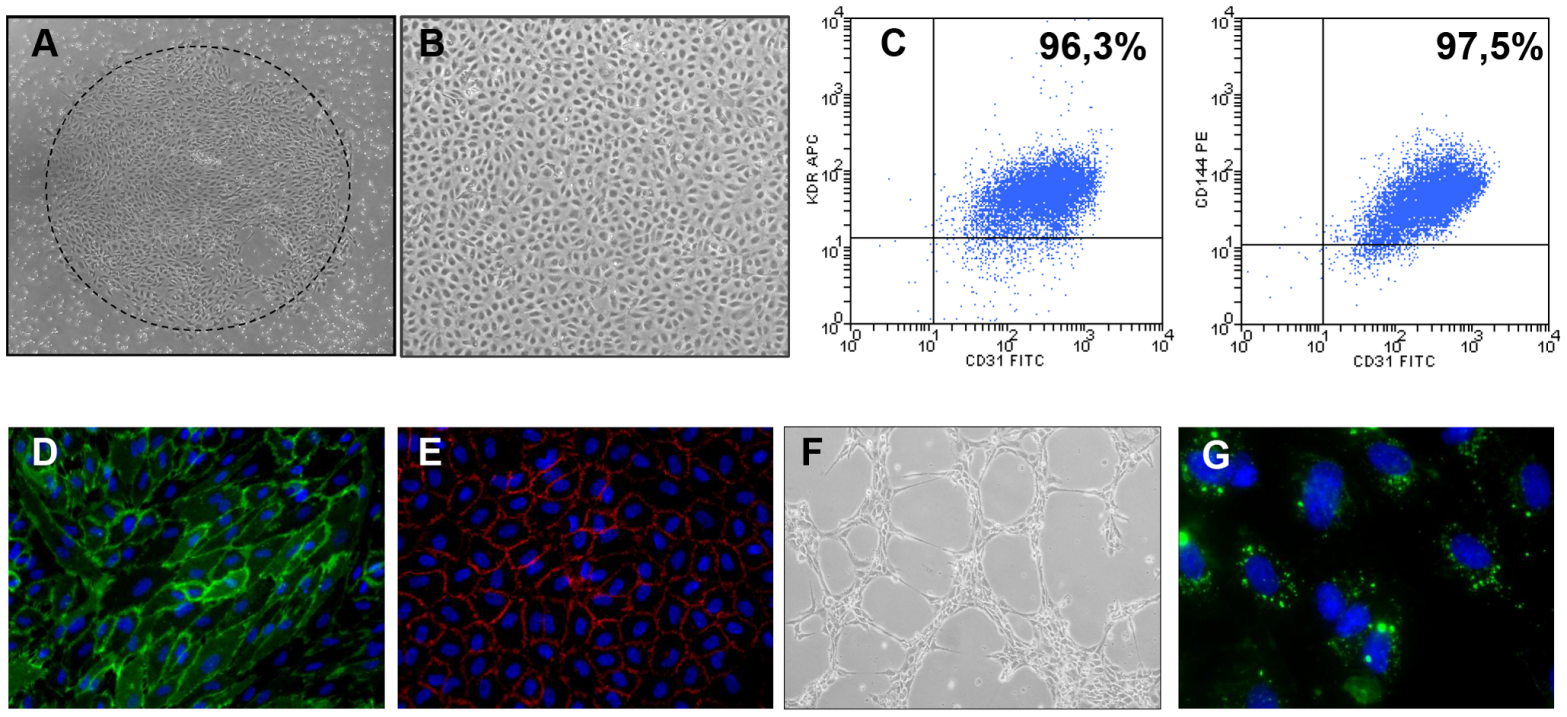
EPDC’S phenotypical and functional characterization. (**A**) Primary colony’s phase contrast micrograph. (**B**) EPDC’s monolayer at passage 2. (**C**) Expression of specific endothelial markers (KDR, CD31/PECAM and CD144/VE-CAD) by flow cytometry. (**D**) CD31, (**E**) CD144 immunofluorescence staining. (**F**) Vascular-like network structures after 4 hours on Matrigel. (**G**) Characteristic diacetylated low-density lipoprotein incorporation.

Cord blood EPCs-derived endothelial cells EPDCs [11] also described as ECFC [12] formed primary colonies (Fig. 1A) that appeared between days 8 and 12 and reached confluence at day 15–20 (Fig. 1B). At this stage, monolayers of confluent cells displayed the typical morphology of endothelial cells. After 2 passages, EPCs were characterized by flow cytometry for expression of specific endothelial markers such as KDR and CD144 (Fig. 1C). Most cells expressed these specific cell surface endothelial markers. Immunofluorescence microscopy showed characteristic CD144 and CD31 membrane expression on most cells of the monolayer (Fig. 1D and 1E). We then expanded these EPDCs during 2 additional passages to confirm they cells maintain features of functional endothelial cells. When EPCs were cultivated on Matrigel, they formed typical vascular-like network structures after 4 hours. Moreover, they showed uptake of diacetylated low-density lipoprotein (LDL) (Fig. 1G). These results confirmed that EPDCs displayed typical phenotypic and functional properties of endothelial cells.

### 2) BBB Specialization

#### a. Functional characterization

**Figure 2:**
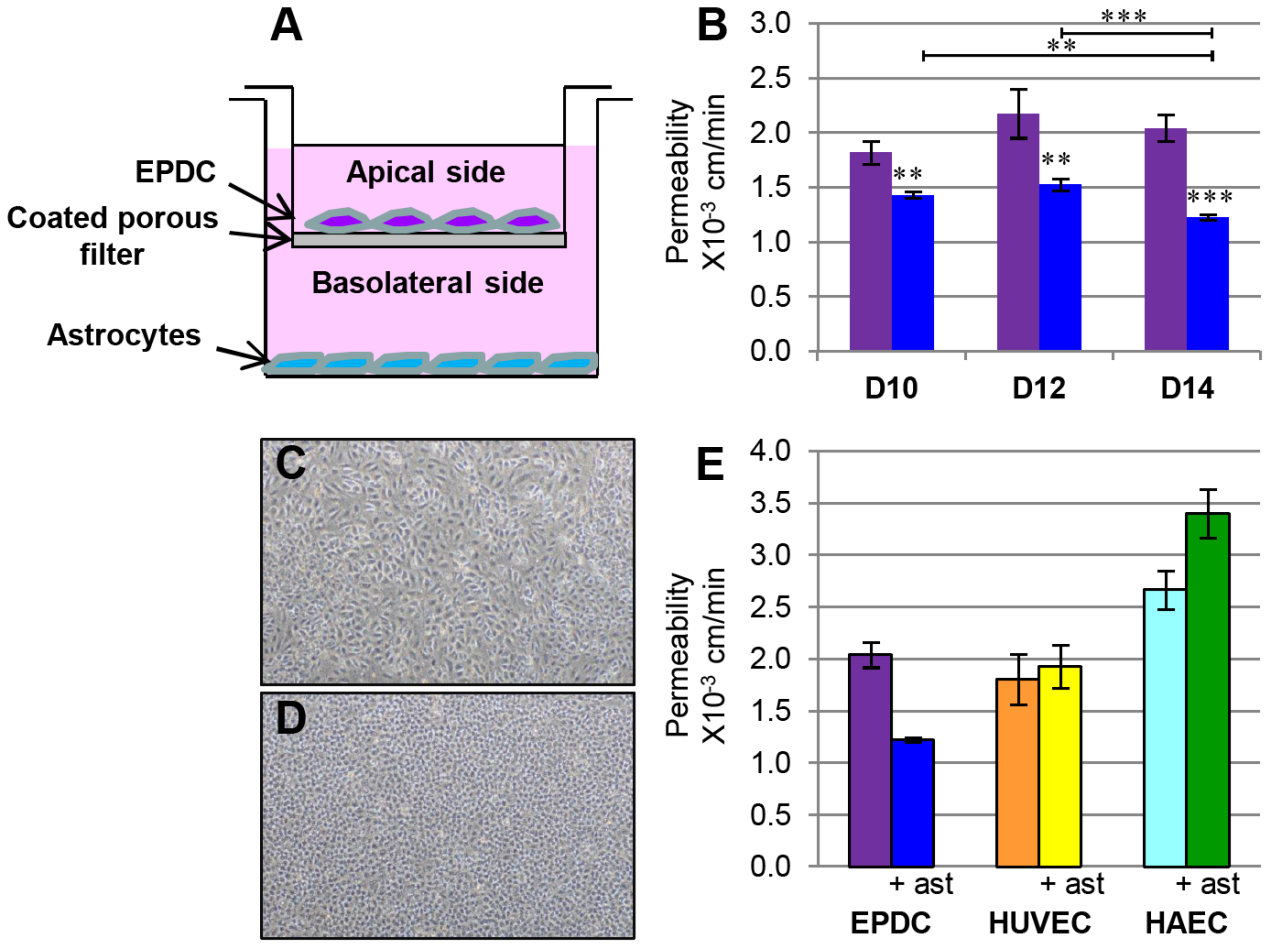
Barrier properties of specialized EPDCs. (**A**) Schematic representation of EPDCs in vitro two-compartment differentiation BBB model. (**B**) Time course of transendothelial permeability for the water soluble small fluorescent marker LY in EPDCs cultured alone (purple) or with astrocytes (blue). (**p<0.001, ***p<0.0001; EPDC permeability measures were performed on 5 independent experiences each individual experience was performed in triplicate). Confluent EPDC’s monolayers cultured alone (**C**) or with astrocytes during 14 days (**D**). (**E**) LY permeability measurement for EPDCs, HUVECs and HAECs cultured alone (left) or with astrocytes (right) (HUVEC and HAEC permeability measures were performed on 2 independent experiences and each individual experience was performed in triplicate). Scale bar: SEM.

EPDCs are stem cell derived endothelial cells that have not yet acquired a specialized phenotype, resembling in that aspect embryonic angioblasts that acquire their specific functional features through induction with organ-specific local signals. For brain tissue, interaction of brain microvascular endothelial cells with astrocytes and pericytes is important to induce BBB specification. To mimic these environmental stimuli in culture, we have developed a two-compartment culture system: EPDCs at passage 2 were seeded onto collagen/fibronectin-coated polyester inserts with rat astrocytes in the lower compartment (Fig. 2A). Co-cultures were then grown in complete endothelial medium until cells reached confluence; EPDCs and astrocytes were then switched to a less proliferative medium up to 14 days to favor maximal “education” in co-culture.

One of the major characteristics of BBB endothelial cells is their strictly limited paracellular permeability (Pe) to hydrophilic compounds, due to their intercellular tight junctions (TJ), comprising proteins such as Zonula Occludens (ZO-1), claudin-5 (CL5) and occludin (OCCL), in addition to adherens junctions (AJ) that contain proteins like VE-cadherin. Permeability was assessed by measuring the passage of a water soluble small fluorescent marker (Lucifer Yellow (LY): MW 457 Da) through confluent monolayers of EPDCs. We identified an optimal time window between day 10 and day 14 of co-culture, when Pe exhibited the lowest values (Fig. 2B). During this period, at each point of the kinetics, the presence of astrocytes significantly decreased the Pe value, as compared to EPDCs cultured alone. Moreover, we showed the lowest permeability (1.23×10^−3^ cm/min) was observed at 14 days of co-culture, which correlates well with the morphology of EPDC at this time (Fig. 2D). Indeed, after 14 days of culture, the morphology of EPDCs co-cultured with astrocytes was different from that of EPDCs cultivated alone. Co-cultured EPDCs appeared smaller and the monolayer appeared tighter than with EPDCs alone (Fig. 2C and 2D). However, beyond 14 days, endothelial cells begin to suffer and to degenerate morphologically, and some of them detach from the filter.

Using hCMEC/D3 cells as an available reference BBB model, we measured permeability to LY which was similar to that observed in the EPDC based model (1.4×10^−3^ cm/min).

To check the robustness of our model, we performed the same BBB-instruction protocol using mature endothelial cells of venous or arterial origin, HUVECs and HAECs (human aortic endothelial cells). In order to avoid cell detachment, LY permeability of HUVECs and HAECs was measured at day 10 of culture. We showed that HUVECs’ permeability, alone or co-cultivated with astrocytes, was equivalent to that of EPDCs cultured alone, whereas permeability of HAECs was higher (Fig. 2E). In contrast to EPDCs, co-culture with astrocytes did not improve permeability values for HUVECs and HAECs. This result indicates that EPDCs, in contrast to HUVECs or HAECs, are specifically responsive to the instructive induction of astrocytes. However, as for the hCMEC/D3 cell line, we measured low TEER values (inferior to 60 Ω/cm2, data not shown).

#### b. Phenotypic characterization

**Figure 3:**
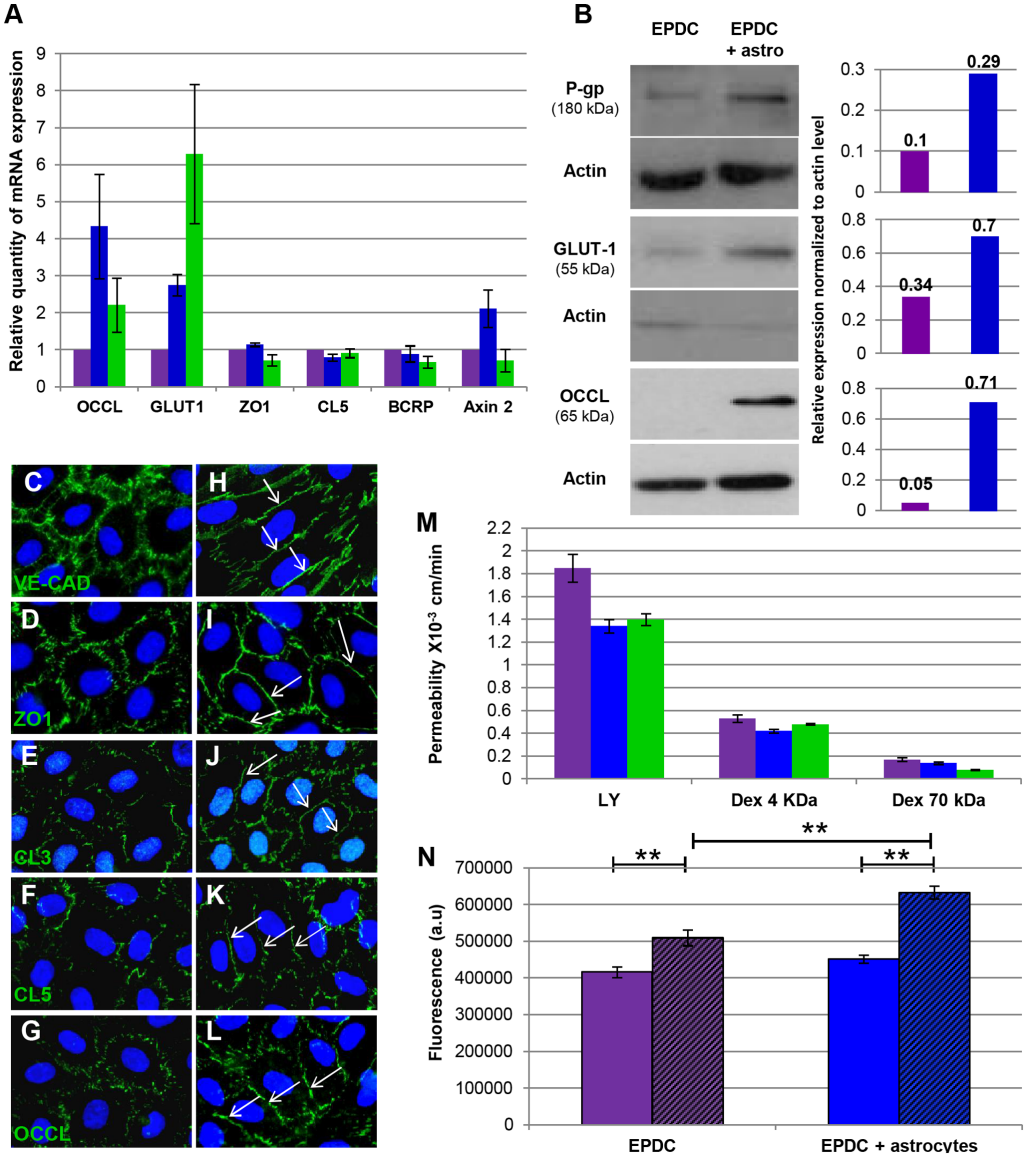
Specialized EPDC’s BBB characteristics. (**A**) Quantitative RT-PCR expression analysis of specific BBB genes at day 14 of culture, in EPDCs alone (purple), with astrocytes (blue) and hCMEC-D3 (green) (qRT-PCR were performed on EPDCs RNA isolated from 6 BBB differentiation independent experiences, each individual experience was performed in triplicate). (**B**) Western blot of P-gp, GLUT-1 and OCCL in EPDCs alone (purple) or with astrocytes (blue) at day 14. Densiometric quantification was performed for each immunoblot using ImageJ software. (**C**–**L**) VE-CAD, ZO1, CL3, CL5 and OCCL immunofluorescence staining in EPDCs alone (**C**–**G**) or with astrocytes (**H**–**L**). Arrows show continuous junctions. (**M**) Permeability for LY (0.457 kDa) and Dextran-FITC (4 and 70 kDa) in EPDCs alone (purple) or with astrocytes (blue) and in hCMEC/D3 (green) (EPDC permeability to dextran-FITC molecules was performed once in triplicate). (**N**) Accumulation of Calcein into EPDCs alone or with astrocytes, in the presence (hatched area) or absence of Verapamil (**p<0.001; a.u: arbitrary units). Scale bar: SEM.

To validate the BBB phenotype of EPDC, we assessed the expression of barrier specific TJ proteins (CL5, ZO-1 and OCCL) and transporters (GLUT1, BCRP and P-glycoprotein: P-gp) by quantitative RT-PCR (Fig. 3A). *OCCL* expression was increased when EPDCs were co-cultured with astrocytes for 14 days. In the same way, *GLUT1*, the primary transporter of glucose present on the BBB, showed an increased expression in the astrocyte co-cultures. Although the expression of *ZO-1*, *CL5* (TJs proteins) and *BCRP* transporter were unchanged in EPCs cultured alone or with astrocytes, we noticed that their levels of expression were similar to that observed in hCMEC/D3 cells. Overexpression of Axin-2 in co-cultured EPCs suggested that the canonical Wnt/β-catenin pathway is activated in this model, in line with the recent demonstration that this pathway is required for BBB formation during development. Moreover, in terms of the expression of P-gp, an active efflux transporter involved in BBB function, we detected by qPCR a very low level of expression as compared to the hCMEC/D3 cells (data not shown). We thus investigated P-gp protein expression by western blot and showed an increased protein level in EPDCs co-cultured with astrocytes compared to EPDCs cultured alone (Fig. 3B). Moreover, western blot experiments confirmed higher expression of GLUT1 and OCCL proteins in EPDCs co-cultured with astrocytes.

P-gp activity was evaluated by a Calcein uptake assay: as described in the Materials and Methods Section, the cell-permeant, non-fluorescent dye Calcein-AM is converted to the fluorescent Calcein, a P-gp substrate, by intracellular esterases. Here (Fig. 3N), in the presence of Verapamil, a P-gp inhibitor, we observed a significantly increased intracellular Calcein level in EPDCs, revealing the activity of the efflux transporter P-gp. More interestingly, this increase was stronger in EPDCs co-cultured with rat astrocytes (1.40-fold increase) than in EPDCs cultured alone (1.23-fold increase): this observation extends the above result by establishing that P-gp activity in EPDCs is enhanced by co-culture with astrocytes. For comparison, in hCMEC/D3 reference cells, the Verapamil-induced increase in the intracellular Calcein was 1.80-fold.

AJ and TJ protein expression after 14 days of culture was assessed by immunofluorescent staining. Co-cultured EPDCs showed a more continuous expression of VE-cadherin, ZO-1, claudin-3 (CL3), CL5 and OCCL at cell-cell contact (Fig. 3D–L), typical of BBB endothelial cells, whereas EPDCs cultured alone showed a more diffuse and less continuous staining (Fig. 3C–K); this observation suggests that endothelial cell-cell junctions undergo maturation in the presence of astrocytes.

As mentioned above, the extremely low permeability of the BBB *in situ* is directly related to the existence of TJs between cerebral endothelial cells which restrict the passive diffusion of polar molecules from blood to brain tissue in a size-selective manner. We tested the permeability of this EPDC-model to standard fluorescent polar molecules of increasing molecular masses (LY: 457 Da, FITC-Dextran 4 kDa and FITC-Dextran 70 kDa). Our results indicated (Fig. 3M) that, as expected, the permeability of human EPDCs was inversely related to the mass of the polar compound tested. Co-culture with rat astrocytes significantly reduced the LY permeability of EPDCs to a similar level as the reference hCMEC/D3 cell line. The low permeability to large molecules of Dextran of the EPDC monolayer did not allow for the detection of any significant further reduction by co-culture with rat astrocytes. This phenotypic and functional characterization indicates that EPDCs can be educated by glial cells to express several BBB markers, suggesting that human cord blood EPCs constitute a previously unrecognized source of cells that could ultimately lead to new *in vitro* models of human BBB.

### 3) Arterial Specialization

#### a. Expression profile of HUAECs and EPDCs compared to HUVECs

It is now established that a molecular imprinting of arterial and venous identities exists prior to the establishment of blood circulation. Arterial and venous specific markers have been identified, such as the Ephrin-B2 ligand (EFNB2), specifically expressed in the arteries, and its receptor, Ephrin-B4 (EPHB4), more restricted to venous endothelial cells.

**Figure 4:**
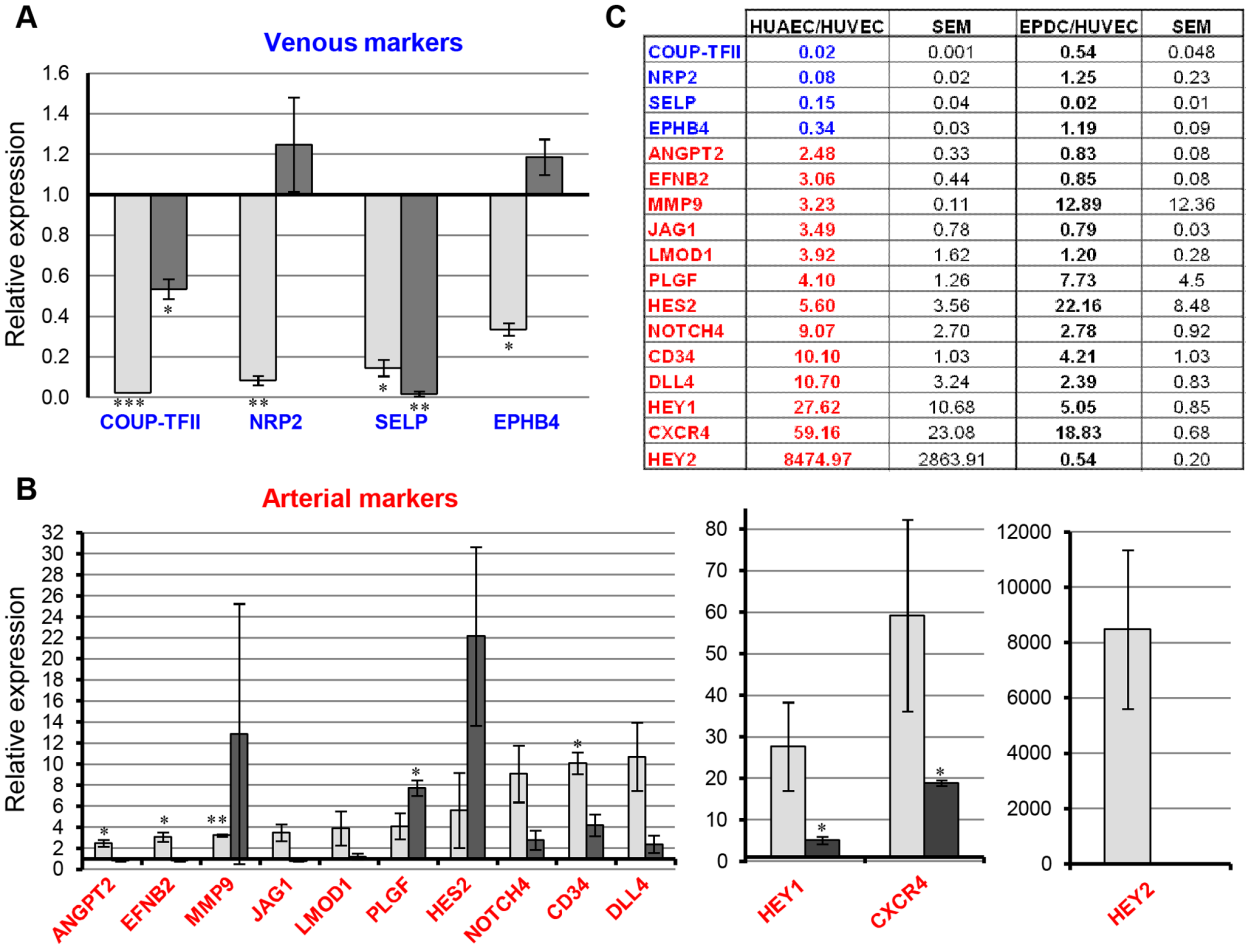
Gene expression profile from HUAECs and EPDCs compared to HUVECs. (**A**) Sixty genes were analyzed using TaqMan® microfluidic cards on early passages HUAECs and EPDCs. Figure 4 shows selection of 17 selected genes confirmed by Gene Expression Assays. Results were expressed as the ratio of expression between HUAECs vs HUVECs (light grey) and EPDCs vs HUVECs (dark grey). (**A**) Expression of venous (blue) and (**B**) arterial (red) markers (scale bars: SEM). (**C**) Table of numeric values. (*p<0.05, **p<0.001, ***p<0.0001) (qPCR expression analysis were performed at least 3 times, on independent cells batches and each time in triplicate).

In order to have access to a specific pattern of arterial and venous specific genes, we screened the expression of sixty selected genes using TaqMan® microfluidic cards on HUAECs and HUVECs. We also established the native gene expression profile of EPDCs under standard culture conditions. This expression profile was performed on early passages because arterial specialization markers decreased with passage number in the absence of flow conditions during culture. HUVECs and HUAECs each showed distinctive mRNA expression patterns. We confirmed the expression of several genes already known to have arterial/venous specificity, such as *EFNB2 and* Notch signaling components (Fig. 4) and identified new arterial markers.

Indeed, expression of six genes appeared to be up-regulated in HUAECs (Fig. 4B and 4C). The *CXCR4* (C-X-C chemokine receptor type 4) gene was expressed almost 60 times more in HUAECs than in HUVECs. We also found an increased expression of *CD34* cell surface marker in HUAECs. This molecule is selectively expressed on human hematopoietic progenitor cells but also on vascular endothelial cells.

*PlGF* (*Placental Growth Factor*) belongs to the family of the vascular endothelial growth factor members. Our results showed that this gene was up-regulated in HUAECs compared to HUVECs. We also observed an increased level of expression of *LMOD1* (*Leiomodin 1*) in HUAECs as compared to HUVECs, which correlated with the protein expression in mouse aorta [34]. *MMP9* (Matrix Metalloproteases 9), which degrades the extracellular matrix to allow for endothelial sprouting, is essential for angiogenesis. HUAECs expressed this gene almost 3 times more than did HUVECs. Lastly, we identified *ANGPT2* as an arterial marker in our model.

Well known venous markers have been confirmed in our model, such as *COUP*-TFII, *NRP2* and *EPHB4* (Fig. 4A and 4C). We also identified a new venous marker, *SELP* (Selectin-P), which belongs to the family of the cellular adhesion molecules. *SELP* was significantly decreased in HUAECs as compared to HUVECs. This protein mediates the interaction of activated endothelial cells with leukocytes.

Expression of these new arterial and venous markers has been investigated in our EPDC-derived arterial model.

Analysis of gene expression in EPDCs showed an intermediate profile between HUAECs and HUVECs. Indeed, expression of *COUP-TFII* and *SELP* venous markers was roughly similar to that in HUAECs (Fig. 4A and 4C). In contrast, venous markers *NRP2* and *EPHB4* were expressed at levels close to that of HUVECs. Analysis of arterial marker expression showed that some genes, such as *PLGF*, *HES2*, *CD34* or *CXCR4* were expressed at levels close to that of HUAECs, whereas expression of *ANGPT2*, *EFNB2*, *JAG1*, *LMOD1* and *HEY2* was closer to HUVECs (Fig. 4B and 4C). In this context, we decided to assess whether EPDCs, which in steady state culture conditions presented an intermediate arterio-venous phenotype, could be differentiated to an arterial phenotype by inductive culture conditions.

#### b. Arterial specialization of EPDCs

**Figure 5:**
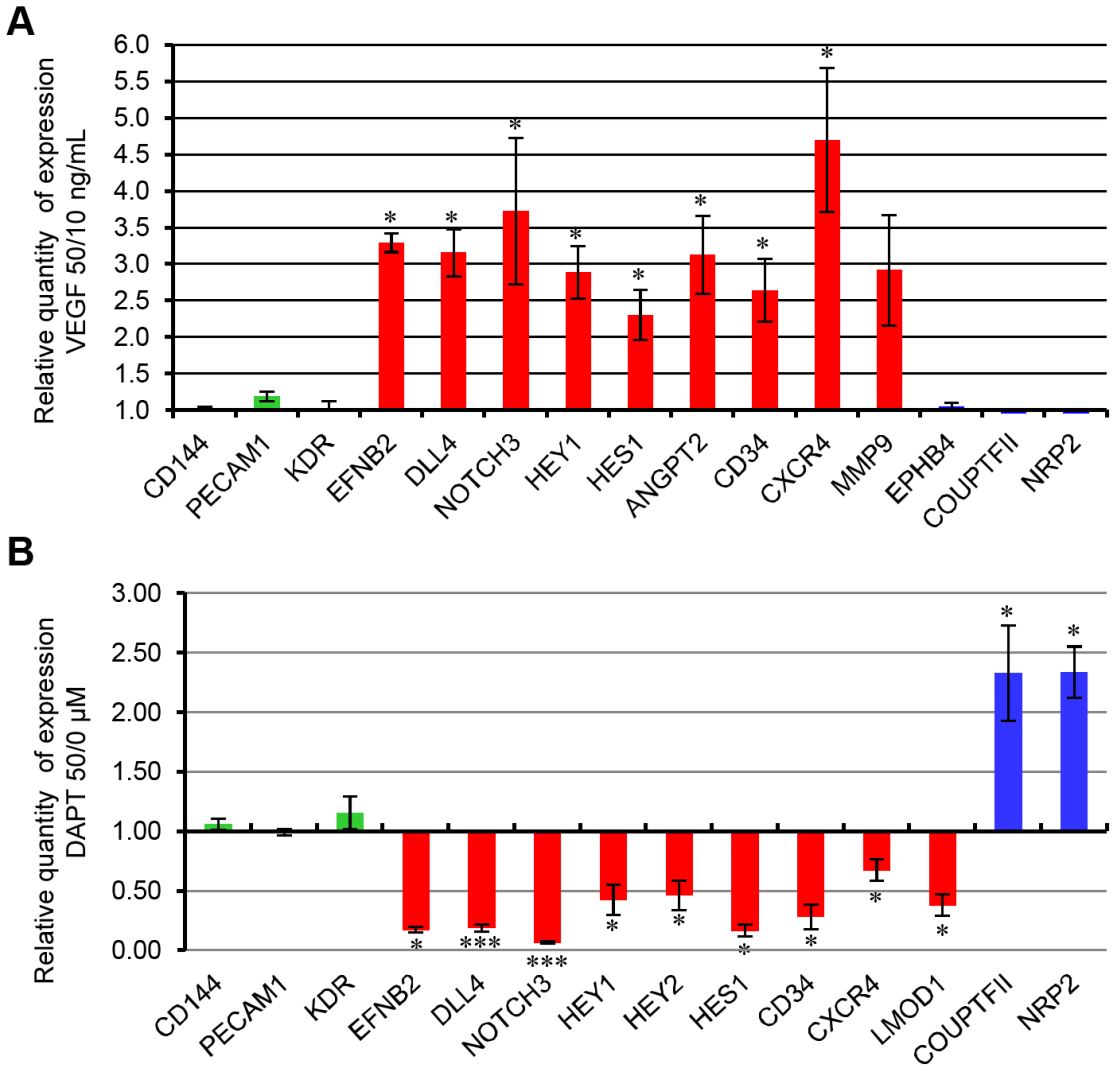
qRT-PCR Expression analysis of arterio-venous genes in EPDCs under arterial and venous inductive conditions. (**A**) Early passages EPDCs were cultured in high (VEGF 50 ng/ml) or low (VEGF 10 ng/ml) VEGF concentration conditions and then, expression of arterio-venous markers were analyzed. Results were expressed as the ratio of expression between high and low VEGF concentration culture conditions. (**B**) High VEGF early passages EPDCs were cultured under 50 µM DAPT, a Notch signaling inhibitor, and then, expression of arterio-venous markers were analyzed. Results were expressed as the ratio of expression between 50 µM and none DAPT conditions (green: endothelial, red: arterial, bleu: venous markers). (Scale bars: SEM, *p<0.05, **p<0.001, ***p<0.0001) (qPCR expression analysis were performed at least 3 times, on independent cells batches and each time in triplicate).

The goal of this part of work was to demonstrate that EPDCs can be specialized into an arterial phenotype by using a simple and reproducible culture method. Several studies have shown that VEGF and Notch signaling were strongly implicated during arterial specification. We thus treated EPDCs with various concentrations of VEGF as early as at passage 1 after EPDC colonies appeared. EPDCs were grown for one or two passages under these conditions and then analysis of the expression of arterial and venous markers was performed. As shown in figure 5A, expression of venous markers detected by qRT-PCR was not modified by the presence of high concentrations of VEGF in the culture medium. In contrast, we noticed that expression of most canonical arterial genes, such as *EFNB2* and *Notch* signaling components was significantly up-regulated. Expression of the new arterial markers identified in HUAECs cultures, *ANGPT2*, *CD34* and *CXCR4* (except *PLGF*) was also increased in EPDCs treated with higher concentrations of VEGF. To confirm that this VEGF-dependent induction of arterial genes was directly related to the activation of Notch signaling in EPDCs, we added the Notch inhibitor DAPT with EPDCs from passage 1, in the presence of high amount of VEGF (50 ng/mL). Indeed, DAPT decreased the expression of arterial markers and increased that of the venous markers *COUP-TFII* and *NRP2*. Among all the genes whose expression was increased by VEGF treatment and decreased with DAPT, we could identify *DLL4*, a ligand of Notch signaling, the receptor *NOTCH3* and 3 Notch effectors, *HES1*, *HEY1* and *HEY2*. These results confirmed the strong implication of the Notch pathway in this EPDC specialization model. Moreover, the decreased expression of all arterial markers observed with DAPT treatment in presence of a high concentration of VEGF confirmed that Notch signaling was involved downstream of VEGF in the arterial specialization.

**Figure 6:**
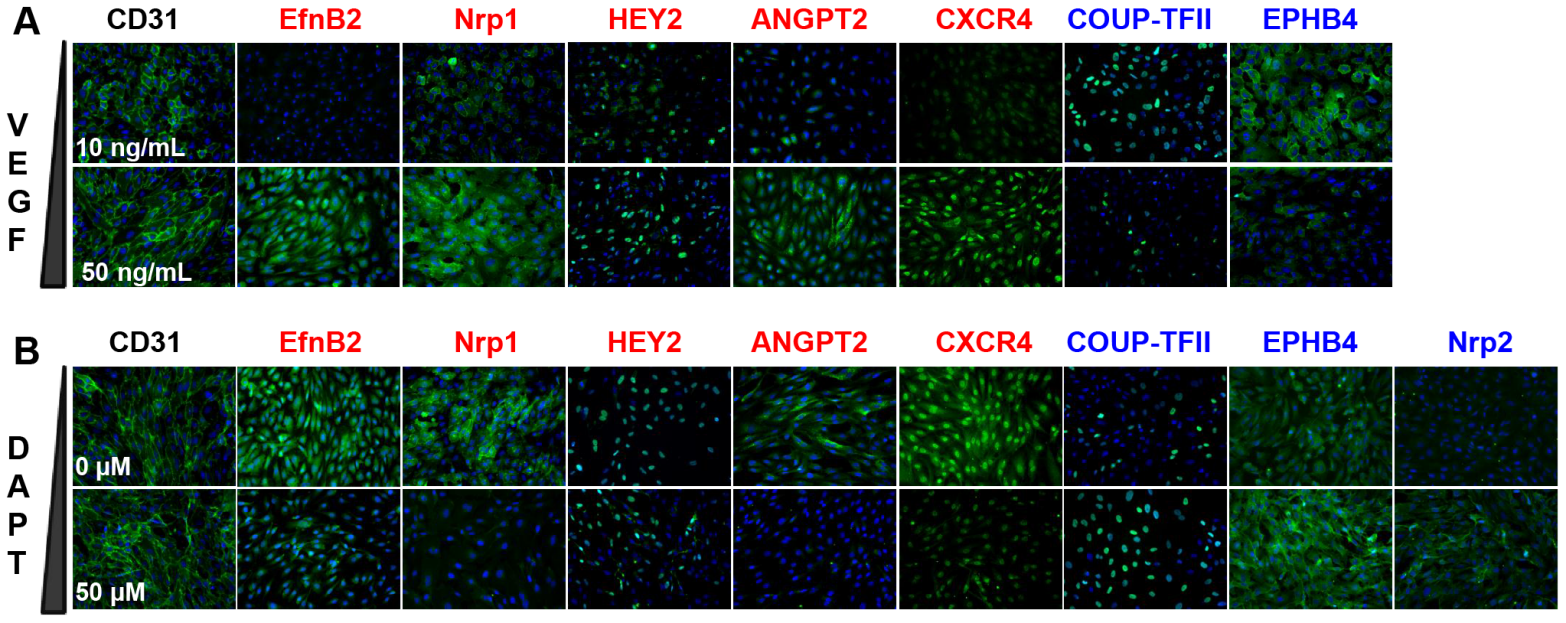
Immunofluorescence analysis of arterio-venous markers in EPDCs under arterial and venous inductive conditions. (A) EPDCs were cultured under high or low VEGF concentrations. (B) High VEGF early passages EPDCs were cultured with 50 µM DAPT (red: arterial markers, bleu: venous markers).

These results were confirmed at the protein level. Indeed, immunofluorescence assays showed that expression of arterial markers, such as EFNB2, Nrp1, HEY2, ANGPT2 and CXCR4, was up-regulated with high VEGF concentrations (Fig. 6A). Although no variation of venous transcripts was observed by qRT-PCR, extinction of venous markers COUP-TFII and EPHB4 in the presence of higher VEGF concentrations was confirmed by immunostaining. We also demonstrated that DAPT treatment repressed arterial marker expression such as EFNB2, HEY2, and CXCR4. Two other arterial genes, *NRP1* and *ANGPT2*, which variation of expression was not evidenced by qRT-PCR analysis, appeared clearly repressed in immunofluorescence experiments (Fig. 6B). Finally, we noted that upon Notch inhibition, the venous markers COUP-TFII, EPHB4 and NRP2 were up-regulated. Taken together, these results demonstrated that early passage EPDCs cultured under VEGF-inductive conditions could adopt a Notch dependent-specialized arterial phenotype, even in absence of shear stress.

## Discussion

EPDCs display the morphology and phenotype of endothelial cells but their functional features indicate that although these cells have undergone some differentiation steps, they still exhibit properties of immature cells. In this study, we have demonstrated that early passage EPDCs display significant plasticity, when submitted to appropriate stimuli, being able to acquire, some distinct characteristics of paradigmatic specialized endothelia: brain or arterial endothelium.

To induce a phenotypic change towards BBB specialization, we have developed an *in vitro* model where EPDCs at passage 2 were grown on culture inserts and co-cultured with rat astrocytes, in the lower compartment. To assess the extent of this phenotype change, we tested several key properties such as the paracellular permeability of the endothelial monolayer to hydrophilic compounds. The restricted permeability observed with EPDCs co-cultured with astrocytes (1.23×10^−3^ cm/min) correlated with staining of intercellular TJ proteins, and was only observed in co-culture conditions. These results are similar to those obtained with the hCMEC/D3 cell line, an available *in vitro* model of the human BBB [20]–[22] which we used here as a reference.

Moreover, we tested commercially available human astrocytes in co-culture with EPDCs, but these cells failed to induce any significant BBB specialization of EPDCs, as compared to rat astrocytes. Further experiments would be required to definitively assess whether human astrocytes may be used successfully in co-culture with EPDCs. Nevertheless, co-culture models of endothelial cells from human [35] or bovine [17] origin and rat astrocytes have been successfully developed by other groups. These observations suggest that putative species differences are unlikely to impede the establishment of new valuable in vitro models of the BBB.

One of the limitations of our model remains the low TEER measured, similar to the hDMEC/D3 cell line, which nevertheless presently constitutes a useful and widely used model of the human BBB. However, we observed with EPDCs a very low permeability to passively diffusing polar compounds (4 kDa or 70 kDa Dextrans) and a reduced permeability to the low molecular weight compound LY (400 Da) in co-culture with rat astrocytes. We also showed that expression of the TJ protein *OCCL,* the glucose transporter *GLUT-1* and the active efflux transporter P-gp, three markers of the BBB, were significantly increased in co-cultured EPDCs. Finally, we report that P-gp activity, as assessed by Verapamil-induced increase in the Calcein intracellular level, was significantly enhanced by co-culture with rat astrocytes.

Although we did not detect, by qPCR, significant differences in the expression of *ZO1*, *CL5* and the *BCRP* transporter between EPDCs cultured alone and co-cultured with astrocytes, we observed that their levels of expression were similar to those of control hCMEC/D3 cells. In line with the recent demonstration that the canonical Wnt/β-catenin pathway is implicated in BBB induction [36], high expression in co-cultured EPDCs of *AXIN*-2, a primary target gene of the Wnt/β-catenin pathway suggested that this essential pathway was indeed activated in ‘educated’ EPDCs. Moreover, immunofluorescence staining of AJ and TJ revealed a typical labeling of BBB endothelial cells. CL3, CL5 and OCCL staining appeared even more continuous at cell-cell contacts compared to hDMEC/D3 [21]. Taken together, these results showed that co-culture of early passages EPDCs with astrocytes induced a phenotypic change towards a BBB phenotype. As this work was being completed, Lippmann et al. independently reported the derivation of BBB endothelial cells from human pluripotent stem cells [35]. Using a completely different cellular model, this study elegantly shows that still immature endothelial cells can further differentiate towards a BBB phenotype under appropriate culture conditions and that contact with neural cells is crucial for this specialization. This robust specialization model, which includes well-organized TJs, expression of nutrient transporters, polarized efflux transporter activity and a high TEER is based on genetically reprogrammed cells. The main advantage of this model is that it leads to an abundant source of differentiated BBB cells, to perform drug screening for example. However, this model has a number of limitations related to the use of these pluripotent cells, which are genetically reprogrammed. Moreover, epigenetic modifications, which persist after reprogramming, would impact on the application of these cells in basic research or drug development [37], [38]. The model proposed in our study, although presenting a less complete BBB phenotype, is still the first proposed model using primary normal human endothelial progenitors, which could ultimately lead to a new in vitro model of the human BBB. The large amount of early passage EPDCs obtained from umbilical cord blood allows appropriate conditions to perform drug testing. Moreover, cord blood, an accessible source for EPC, gives access to the study of inter-individual biological variabilities, which constitutes an important asset for pharmaceutical studies. In any event, both models may constitute original tools for producing human *in vitro* models for drug testing.

To extend the concept of specialization of early passage EPDCs, we assessed their response to culture conditions leading to an arterial specialization. Indeed, after identification of a new set of arterial markers differentially expressed by HUAECs and HUVECs, we showed here that high concentrations of VEGF induced early passage EPDCs to express a series of arterial markers: VEGF-treated EPDCs expressed both classical (*EPHB2*, *DLL4*, *NOTCH3*, *HEY1* and *HES1*) and newly identified (*ANGPT2*, *CXCR4*, *MMP9* and *CD34)* arterial markers.

Although the new genes we identified were not precisely described as arterial markers in other studies, previous reports were consistent with our study. Indeed, expression of PlGF, which is induced by more than 4 fold in HUAECs as compared to HUVECs, was detected in several types of cultured human endothelial cells; PlGF expression, production and secretion was reported to be higher in Human Pulmonary Arterial Endothelial Cells than in HUVECs, supporting the arterial specificity of this marker [39]. We also showed that *CXCR4, CD34* and *ANGPT2* expressions were significantly increased in HUAECs and in EPDCs cultured in VEGF-inductive conditions, compared to HUVECs. CXCR4, which is the receptor of the SDF1 (CXCL12) chemokine, was detected in endothelial cells of the dorsal aorta but not of cardinal veins in the aorta-gonado-mesonephros (AGM) region of E11.5 mouse embryos, confirming an arterial specificity of the gene [40]. Moreover, immunohistochemical analysis of the CD34 antigen expression has shown a strong expression in large vessels of the umbilical cord, especially in arteries [41], which correlates with our results. Finally, a recent study has demonstrated that the *ANGPT2* gene was up-regulated in rat mesenteric microvascular arteries compared to veins [42].

It is well known that VEGF is required for arterial specialization. It has been shown, particularly in zebrafish, that *sonic hedgehog* acts upstream of *VEGF* by inducing the latter’s expression [33]. The Notch signaling pathway is crucial for arterial differentiation by acting downstream of VEGF induction [24], [25]. Indeed, we confirmed here that VEGF-induce arterial specialization of EPDCs depended on Notch signaling. Incubation of VEGF-treated cultures with a Notch inhibitor (DAPT) strongly repressed the expression of most arterial markers and increased that of venous markers. These results indicate that the EPDCs arterial specialization model proposed here involves canonical molecular pathways such as VEGF and Notch signaling, via the Notch3 receptor. Moreover, we showed that culture under flow conditions is not obligatory for establishing an arterial profile, although it is probably required for its maintenance during further passages. Indeed, arterial-venous development and specification in the embryo has been assumed to depend on the influence of fluid mechanical forces [43], [44]. Masumura et al. have demonstrated that shear stress induces an increase in expression of the arterial endothelial marker EphrinB2 in murine ES cells via the VEGF-Notch signaling pathways [27]. Moreover, when cultured EPCs were exposed to shear stress, the gene expression levels of the arterial endothelial markers ephrinB2, Notch1/3, Hey1/2, and ALK1 increased, whereas the gene expression levels of the venous endothelial markers EphB4 and NRP2 decreased [45]. Although Notch1 and Notch4 are known to be expressed in arterial endothelial cells, it appears that Notch3 is the receptor involved in the arterial specialization of EPDCs by VEGF and/or shear stress induction [25], [45]–[47].

In summary, the present study illustrates that EPDCs grown under specific instructive culture conditions can further differentiate towards specialized phenotypes. We propose that EPDCs may now be considered as a promising source of plastic endothelial cells, capable of acquiring distinct tissue specific characteristics which may benefit drug testing and to the development of predictive *in vitro* assays.
